# The role of tenascin-C in tumor microenvironments and its potential as a therapeutic target

**DOI:** 10.3389/fcell.2025.1554312

**Published:** 2025-02-19

**Authors:** Yaran Wang, Xiaohui Wen, Chao Su, Yanyi You, Ziqing Jiang, Qin Fan, Daoqi Zhu

**Affiliations:** The School of Traditional Chinese Medicine, Southern Medical University, Guangzhou, Guangdong, China

**Keywords:** tumor microenvironment, tenascin-c, immune modulation, cancer biomarker, radiation microenvironment, cancer therapeutics

## Abstract

The tumor microenvironment (TME) plays a pivotal role in cancer development and progression, and comprises various cellular and non-cellular components that interact with tumor cells. Tenascin-C (TNC) is an extracellular matrix glycoprotein that is widely expressed in the cancer stroma and influences critical processes, such as cell adhesion, migration, and immune modulation. This review examines the multifaceted roles of TNC in different TMEs, including the mechanical, immune, and metabolic microenvironments, as well as the radiation microenvironment (RME). In the context of the mechanical microenvironment, TNC actively participates in extracellular matrix remodeling, thereby facilitating tumor invasion. Notably, TNC exhibits immunosuppressive effects on T cells and promotes the recruitment of immunosuppressive cells within the immune microenvironment. Furthermore, TNC is implicated in the tumor hypoxia response, glucose metabolism reprogramming, and regulation of pH balance, underscoring its role in the metabolic microenvironment. Intriguingly, TNC also influences radiosensitivity within RME. This review also explores the potential of TNC as a biomarker for cancer prognosis and as a target for therapeutic interventions. By integrating recent advances in single-cell sequencing and spatial omics, we propose innovative strategies for leveraging TNC in personalized cancer therapy. Future research directions are discussed, focusing on distinct isoforms of TNC, their interaction networks, and their roles in radiotherapy efficacy. This comprehensive analysis underscores the importance of TNC in understanding tumor dynamics and improving cancer treatment outcomes.

## 1 Introduction

The tumor microenvironment (TME) consists of non-cancerous cells, vasculature, lymphatic structures, nerves, extracellular components, and metabolites located within and around the tumor site. TME development begins when malignant cells with cancer-causing mutations attract nearby normal cells and release various intercellular signaling molecules. This process creates a favorable environment that gradually adapts to facilitate cancer cell growth, movement, and defense against external threats (Jin and Jin, 2020). Tenascin-C (TNC) is an extracellular matrix glycoprotein widely and abundantly expressed in the cancer stroma (Chen et al., 2024). TNC interacts with multiple receptors through its unique six-armed structure, influencing biological processes, such as cell adhesion, migration, and proliferation, thereby playing a significant role in cancer initiation and progression (Cheng et al., 2021; Yilmaz et al., 2022). TNC is linked to the mechanical, immune, metabolic, and radiation microenvironments (RMEs) of the tumor, and affects tumor progression.

Recent advancements in research techniques have revealed the mechanisms of action of TNC in TME. A thorough understanding of the role of TNC in various TMEs is crucial for understanding the underlying patterns of cancer development and progression, as well as for identifying new prognostic indicators and treatment targets. Here, we review the latest research on TNC in mechanical, immune, metabolic, and RMEs, and discuss its potential as a cancer biomarker.

## 2 Basic characteristics of TNC

### 2.1 Structure and functions of TNC

The *human Tenascin-C* (*hTNC)* gene is situated on the long arm of chromosome 9, specifically at band 33 of region 1, encompassing approximately 98.6 kb and consisting of 32 exons. TNC is a well-preserved hexameric glycoprotein with a molecular structure comprising an oligomerization domain (TA), 14 1/2 EGF-like (EGFL) repeats, 17 fibronectin type III (FNIII) repeats, and a C-terminal fibrinogen-like globular (FBG) domain (Dhaouadi et al., 2024). Between the 5th and 6th FNIII domains of the human protein, there are nine alternatively spliced FNIII domains (A1–A4, B, AD1, AD2, C, and D). The inclusion of additional FNIII domains enlarges the TNC protein, resulting in a long TNC isoform, whereas the short isoform comprises only the constant FNIII domains 1–8. The short TNC isoform is predominantly found in normal tissues. However, certain long TNC isoforms are expressed in tumors (Dhaouadi et al., 2024). For example, the alternatively spliced FNIII domain AD1 is found in human glioblastoma, neuroblastoma, and osteosarcoma tumor cells, but not in healthy human lung fibroblasts or human umbilical vein endothelial cells (Giblin and Midwood, 2015). TNC consists of four distinct domains that allow it to interact with more than 25 different molecules, including EGF receptors, platelet-derived growth factor, fibroblast growth factor (FGF), transforming growth factor-β (TGFβ) family members, FBG-domain integrins, receptor-type tyrosine protein phosphatase zeta, and Toll-like receptor 4 (TLR4). Through these interactions, TNC influences various processes, such as cell proliferation, migration, and adhesion; focal adhesion; neurite growth and enhancement; and the maintenance of inflammatory states (Giblin and Midwood, 2015; Lowy and Oskarsson, 2015). The following research provides a detailed examination of some of the key functions of TNC mentioned above. During muscle regeneration, myofibers undergo necroptosis, releasing TNC. Through its N-terminal assembly domain and EGF-like domain, TNC activates the EGFR signaling pathway in muscle stem cells (MuSCs), promoting their proliferation and thereby aiding in muscle repair (Zhou et al., 2020). Cai and colleagues further elucidated the critical role of TNC in both neurite outgrowth and the maintenance of inflammation. Specifically, in the context of neurite outgrowth, TNC, induced by inflammation and secreted by papillary fibroblasts, interacts with integrin receptors such as α7β1 on the neuronal membrane. This interaction activates the ERK signaling pathway, which, in turn, promotes abnormal branching and elongation of neurites. Regarding the maintenance of inflammation, TNC expression is significantly elevated at inflammatory sites, such as in psoriasis. It binds to surface receptors on immune cells, modulating their activity and function, which exacerbates skin inflammation (Cai et al., 2023).

### 2.2 Distribution of TNC expression

TNC is highly expressed in developing embryos; however, its presence in most healthy adult tissues is minimal and is limited to areas with high cell turnover rates or where tissue remodeling is necessary, such as stem cell niches, the central nervous system, and regions subjected to significant tensile stress (e.g., tendons, ligaments, and smooth muscle fibers). Temporary TNC expression can be observed at numerous tissue injury sites (Zhou et al., 2020). For instance, in the lesioned skin of patients with atopic dermatitis (AD), TNC expression is significantly increased (Mitamura et al., 2023). Spatial transcriptomics and single-cell RNA sequencing analyses have revealed that TNC is highly concentrated in leukocyte-infiltrated areas of AD lesional skin, where it colocalizes with COL18A1+ fibroblasts, a distinct subpopulation characterized by expression of the *COL18A1* gene encoding collagen type XVIII alpha 1 chain protein (Mitamura et al., 2023). In pulmonary diseases, TNC expression is also elevated in basal epithelial cells and fibroblasts of patients with asthma, as well as in Alveolar Type 2 (AT2) cells and endothelial cells of patients with chronic obstructive pulmonary disease (Donovan et al., 2023).

Research has shown that TNC is highly expressed in the inflamed mucosa of individuals with ulcerative colitis (UC) and Crohn’s disease (CD) (Ning et al., 2019). An analysis of renal biopsy samples from patients with tubulointerstitial nephritis (TIN) indicated that TNC emerged during active inflammation and disappeared as healing occurred (Izumi et al., 2020). TNC is also abundant in cancer tissues exhibiting increased cell turnover or tissue remodeling (Giblin and Midwood, 2015; Lowy and Oskarsson, 2015). Previous studies have shown that the deposition of TNC increases in the tumor stroma in most epithelial cancers (Yoshida et al., 2015), such as breast cancer (Nagaharu et al., 2011), ovarian cancer (Steitz et al., 2020), pancreatic cancer (Furuhashi et al., 2023), colon cancer (Fujita et al., 2021b), gastric cancer (Kang et al., 2021), and others.

### 2.3 TNC and tumors

TNC, particularly the long isoform, is crucial in tumors because it enhances their proliferation and migration while suppressing anti-tumor immune responses (Wickman et al., 2024). TNC can modulate the mechanical features of TME, primarily by altering the stiffness of the extracellular matrix (ECM) (Miroshnikova et al., 2016), promoting epithelial-mesenchymal transition (EMT) (Nagaharu et al., 2011; Katoh et al., 2013; Yang et al., 2019; Wu et al., 2023), and interacting with key components of the mechanical microenvironment, such as fibronectin (FN) and cancer-associated fibroblasts (CAFs) (Wu et al., 2023). Various studies have linked TNC to the mechanical microenvironment of tumors, including gliomas (Brösicke and Faissner, 2015), lung cancer (Wu et al., 2023), pancreatic cancer (Chen et al., 2009), and breast cancer (Nagaharu et al., 2011). In prostate cancer, TNC hinders the activation, proliferation, and cytokine production of tumor-antagonizing immune cells, such as T cells, thus impeding the anti-tumor immune response (Jachetti et al., 2015). Similarly, TNC has also been found to be closely associated with the immune microenvironment in other cancer types, including bladder cancer (Gao et al., 2022), oral squamous cell carcinoma (Spenlé et al., 2020), and breast cancer (Huang et al., 2023). In prostate cancer (Qian et al., 2022), neuroblastoma (Xing et al., 2015), low-grade gliomas (Zhang et al., 2022), and esophageal squamous cell carcinoma (Yang et al., 2019), TNC has been closely associated with hypoxic conditions or tumor cell energy metabolism, influencing the tumor’s metabolic microenvironment. Furthermore, TNC has been found to impact the radioresistance of nasopharyngeal carcinoma (Liu et al., 2021).

## 3 TNC and TMEs

As an extracellular matrix protein closely linked to tumor progression, TNC has been extensively investigated in relation to the ECM and mechanical microenvironment. Our investigation primarily focused on TNC’s role in the mechanical microenvironment and its complex interactions with ECM components. Additionally, given the fundamental importance of immune responses in cancer development, we analyzed the intricate relationship between TNC and the immune microenvironment. While TNC’s metabolic interactions can be examined through various perspectives, current research has predominantly concentrated on the link between TNC and hypoxic niches, leaving other metabolic aspects as promising areas for future investigation. We then focused on a recently proposed TME; that is, RME. This environment is intricately connected to the aforementioned TMEs, and its relationship with TNC began to emerge in previous research by our team. Although current research on this topic is limited, the relationship between TNC and RME shows significant research potential.

### 3.1 TNC and the mechanical microenvironment

The mechanical microenvironment is an emerging area of TME research that encompasses intracellular components (such as vimentin, actin, and neurofilaments), extracellular components (such as collagen and fibrin), intercellular signaling molecules (integrins), and stromal cells (fibroblasts) (Jin and Jin, 2020). As an extracellular matrix glycoprotein, TNC is significantly upregulated in processes such as regulating ECM stiffness and EMT, and promoting the formation and maturation of tumor stromal channels (Yoshida et al., 2015), potentially contributing to tumor migration and immune evasion. Glioblastoma Multiforme (GBM) shows increased ECM stiffness compared to diffuse low-grade glioma (LGG), and TNC expression levels are positively correlated with this level of stiffness. Stiffer ECM environments lead to a reduction in miR-203 expression, which in turn enhances the expression of hypoxia-inducible factor 1-alpha (HIF1-α), a key transcription factor involved in cellular responses to hypoxia. HIF1-α then directly activates TNC gene transcription by binding to its promoter. The resulting increase in TNC levels further stiffens the ECM, creating a self-reinforcing cycle. Additionally, research has shown that in xenograft tumor models, where wild-type IDH1-expressing human GBM cells are transplanted into nude mice, reducing TNC expression levels significantly decreases ECM stiffness in tumor tissues and prolongs survival in mice with tumors (Miroshnikova et al., 2016).

TNC is linked with metalloproteinases (MMPs). Studies of the regulation of breast cancer invasion have revealed that specific TNC variants, such as TNC-16 and TNC-14/16, play vital roles in promoting cancer invasion and growth, partly dependent on MMP activity (Hancox et al., 2009). Furthermore, TNC-14/AD1/16, a specific variant of tenascin-C containing exons 14, AD1, and 16 in the variable region, has been shown in the study by Guttery et al. to significantly promote tumor cell invasion and growth, as demonstrated through analyses of breast cancer tissues and cell lines (Guttery et al., 2010). In pancreatic cancer, TNC triggers the JNK/c-Jun signaling pathway, enhancing c-Jun binding to the *MMP9* promoter, thus increasing MMP9 expression levels and activity (Cai et al., 2017). In metastatic lung cancer, TNC interacts with ECM proteins, such as FN, to stimulate CAFs to produce MMP2, thereby remodeling the ECM (Wu et al., 2023). During pancreatic cancer metastasis, TNC secreted by cancer and stromal cells is cleaved by activated MMP2 to form fibrous structures (fTNC) with the help of stromal fibroblasts. These structures facilitate cancer cell migration by diminishing the adhesion between integrin α6β1 and the ECM (Chen et al., 2009).

TNC induces EMT. In esophageal squamous cell carcinoma, high TNC expression levels are observed at the invasive front of the tumor, showing a significant positive correlation with the expression levels of the EMT-related gene, Snail. TNC can also promote EMT through an Akt/HIF1-α-dependent mechanism (Yang et al., 2019). In metastatic lung cancer, TNC activates key transformation-related transcription factors such as Snail homolog 1 (SNAI1), Slug (SNAI2) and Zinc finger E-Box binding homeobox 1/2 (ZEB1/2) that induce EMT (Wu et al., 2023). Katoh et al. discovered that TNC can trigger EMT-like changes in breast cancer cells by binding to integrins αvβ6, αvβ1, and α9β1 (Katoh et al., 2013). Another study suggests that exogenous TNC can induce EMT-like changes in breast cancer MCF-7 cells, characterized by reduced cell-cell adhesion and enhanced migratory ability. Molecular studies have revealed that TNC treatment significantly increases proto-oncogene tyrosine-protein kinase Src(SRC) protein phosphorylation at the Y418 site, with corresponding increases in phosphorylation of the substrate focal adhesion kinase (FAK) at the Y861 and Y925 sites. Further mechanistic investigations showed that both an anti-αv integrin antibody (AV1) and an SRC kinase inhibitor (pp2) effectively inhibit TNC-induced EMT-like changes. Immunofluorescence experiments confirmed the co-localization of phosphorylated SRC and FAK with αv-integrin-positive focal adhesions (Nagaharu et al., 2011). These findings highlight the crucial roles of TNC, integrin αv, and the SRC/FAK signaling pathway in regulating EMT-like changes in tumor cells.

TNC contributes to the development and maturation of tumor stromal channels. These channels are tubular structures that are abundant in ECM proteins and are commonly found in various solid tumors. Fonta et al. revealed that TNC forms a network with FN and collagen fibers within tumor stromal channels. As tumors advance, these channels evolve and the FN fiber tension decreases. This unique environment traps CD8^+^ and M2 macrophages. Without TNC, the channel structure remains, but differs morphologically from that of the wild-type channel (Fonta et al., 2023). TNC aids in the development and progression of tumor stromal channels by establishing a low-tension FN fiber environment that attracts and retains both tumor-suppressing immune cells, such as CD8^+^ T cells, and tumor-promoting immune cells, such as M2 macrophages, potentially influencing tumor spread, metastasis, and immune evasion.

TNC is closely linked to stromal cells, especially CAFs, which are major producers of TNC (Labedz et al., 2024). Recent studies have further supported this concept. For example, Wang et al. found that Caveolin-1 (Cav-1) in exosomes derived from breast cancer cells can stimulate lung fibroblasts to synthesize TNC and other proteins, thereby promoting the deposition of ECM (Wang et al., 2023). Likewise, in the context of corneal wound healing, Fujita et al. suggest that tolerogenic dendritic cells, activated by transforming growth factor-β1 (TGF-β1), can induce fibroblasts to secrete TNC. This effect may occur through the local production of TGF-β1 during the early phase of wound contraction following full-thickness corneal hydrogel transplantation (Fujita et al., 2021a). Interestingly, TNC has been shown to inhibit CAF apoptosis through the activation of the PI3K-AKT pathway (Ni et al., 2017). CAFs are crucial in radiation-induced tissue fibrosis, as they produce excess collagen and other extracellular matrix proteins when activated. TNC contributes to fibrosis via interactions with CAFs. The resulting ECM remodeling creates a barrier that affects oxygen diffusion and worsens hypoxia (Wang et al., 2019), promoting angiogenesis, EMT, and tumor progression (Telarovic et al., 2021).

### 3.2 TNC and the immune microenvironment

Immune cells in tumors can be categorized as tumor-suppressing, tumor-promoting, or controversial (specifically, B cells). Tumor-suppressing immune cells include effector T cells (CD8^+^ cytotoxic T cells and effector CD4^+^ T cells), natural killer cells, dendritic cells, M1-polarized macrophages, and N1-polarized neutrophils. Tumor-promoting immune cells mainly comprise regulatory T cells (Tregs) and myeloid-derived suppressor cells (MDSCs) (Lei et al., 2020).

The impact of TNC on the tumor immune microenvironment is multifaceted. This environment can be reshaped by influencing the function and distribution of various immune cells. TNC exhibits clear immunosuppressive effects on T cells by inhibiting their activation *in vitro* (Lowy and Oskarsson, 2015). Jachetti et al. showed that prostate cancer stem-like cells suppress T cell activation, proliferation, and cytokine production through TNC-α5β1 integrin interactions, inhibiting actin-based cytoskeletal remodeling (Jachetti et al., 2015). Stem-like brain tumor-initiating cells release TNC via exosomes, which then interacts with integrin receptors α5β1 and αvβ6 to suppress the T cell mTOR pathway, thus dampening systemic T lymphocyte immunity in patients (Mirzaei et al., 2018). Radiotherapy may also cause T-cell immunosuppression. Repeated radiation induces persistent type I interferon production and promotes radiation resistance (Jarosz-Biej et al., 2019), and radiation-induced interferons increase PD-L1 expression levels, leading to T cell exhaustion (Shevtsov et al., 2019). However, the relationship between TNC and radiation therapy requires further investigation. TNC also promotes the recruitment of tumor-promoting immune cells, such as Tregs and MDSCs, enhancing their immunosuppressive functions. In a study on LGG, Zhang et al. found increased immunosuppressive cell infiltration and elevated levels of immunosuppressive factors, such as TGF-β and IL10, in the high-TNC subgroup (Zhang et al., 2022). Previous studies have shown that radiation therapy can promote the infiltration of immunosuppressive cells, including Tregs and MDSCs, into TME, hindering anti-tumor immune responses (Lau et al., 2007; Gabrilovich and Nagaraj, 2009; Persa et al., 2015). However, the relationship between TNC and radiation therapy requires further exploration. There are different views regarding TNC and tumor-associated macrophages (TAMs). Some researchers have suggested that the interaction between TAMs and TNC promotes tumor progression, as seen in high-grade serous ovarian cancer, where TAM-derived TNC in the ascites enhances cancer cell migration and progression (Steitz et al., 2020). Others have proposed that this interaction is part of the body’s defense mechanism against tumor cells. In in situ xenografts of human and mouse CD47-homozygous-knockout GBM cells, the loss of CD47 function increases TNC expression levels in tumor cells. This upregulation of TNC triggers the release of pro-inflammatory factors, such as TNF-α, through TLR4-and STAT3-dependent mechanisms in human macrophages, enhancing the recruitment of M2-like TAMs and boosting their phagocytic activity, thereby inhibiting tumor growth (Ma et al., 2019).

### 3.3 TNC and the metabolic microenvironment

#### 3.3.1 The role of TNC in the tumor response to hypoxia

TNC and HIF signaling pathways are essential for regulating the tumor response to hypoxia. Research using neuroblastoma models has shown that TNC-positive cells can transform into tumor-derived endothelial cells (TDECs) under hypoxic conditions. Targeting TDECs intensifies the hypoxic state in tumor tissues, leading to increased expression levels of HIF-2α, a reliable marker of hypoxia in neuroblastoma. This upregulation of HIF-2α enhances the expression of EMT-related genes (CXCL5, IL-6, and FGF-1) and increases the proportion of TNC-positive neuroblastoma progenitor cells (Xing et al., 2015). In LGGs, a significant correlation between TNC and HIF-1α levels has been observed. Extended hypoxia results in a time-dependent increase in HIF-1α and TNC expression levels. TNC colocalizes with the hypoxia marker carbonic anhydrase 9 (CA9) in tumor tissues, indicating its involvement in the tumor response to hypoxia. Patients with high TNC expression levels show elevated hypoxia-related gene scores, including increased expression levels of vascular endothelial growth factor A (VEGFA) and lactate dehydrogenase A (LDHA) (Zhang et al., 2022). In esophageal squamous cell carcinoma, TNC may enhance cancer stem-like properties through the Akt/HIF1α pathway under hypoxic conditions (Yang et al., 2019). Radiation-induced vascular damage can cause tissue hypoxia. HIF, a central regulatory factor in the response to hypoxia, modulates fibrotic processes following radiation therapy (Telarovic et al., 2021). TNC is significantly associated with HIF and participates in the fibrotic process through reciprocal maintenance of CAFs(Ni et al., 2017; Labedz et al., 2024). As fibrosis progresses, tissue hypoxia worsens (Miroshnikova et al., 2016), stimulating angiogenesis and the EMT process (Telarovic et al., 2021) and enhancing tumor invasion and migration. HIF regulates multiple downstream genes that mediate radiation resistance (Telarovic et al., 2021), whereas CAFs activated during fibrosis secrete various factors that promote tumor cell DNA damage repair, further enhancing radiation resistance (Wang et al., 2019). These mechanisms collectively promote tumor progression and create a detrimental cycle that fosters radiation resistance.

The production of reactive oxygen species (ROS) is linked to hypoxia (Gutsche et al., 2016), and increased ROS levels are intricately associated with cancer development, immune responses in tumors, and alterations in TME (Jin and Jin, 2020). A study by Gutsche et al. on inflammatory breast cancer (IBC) revealed that intermittent hypoxia (IH) generates ROS, causing oxidative stress. This activates the NF-κB signaling pathway, enhancing TNC expression in IBC cells and promoting their migration. Interestingly, TNC can also trigger TLR4 expression, which further activates NF-κB, creating a feedback loop in which IH and inflammation mutually reinforce each other *in vivo* (Gutsche et al., 2016). Xing et al. found that elevated ROS levels induced by adiponectin treatment significantly reduced TNC expression levels and increased apoptosis in hepatocellular carcinoma (HCC) cells (Xing et al., 2015). Despite the established connection between TNC, ROS, and oxidative stress, the precise underlying molecular mechanisms remain unclear.

#### 3.3.2 TNC and glucose metabolism reprogramming

Research has indicated a correlation between TNC expression levels and glycolytic metabolism in tumor cells. In prostate cancer cells, TNC expression is significantly positively correlated with the expression levels of key glycolytic enzymes, such as glucose transporter 1 and hexokinase 2 (HK2). Suppressing TNC expression through siRNA decreases glucose uptake and lactate production in the prostate cancer cell lines DU145, PC3, and LNCaP, while substantially decreasing the protein levels of the glycolytic enzymes HK2, LDHA, and pyruvate kinase isozyme M2 (PKM2) (Qian et al., 2022). An investigation has shown that TNC modulates the glycolytic process in prostate cancer cells by activating the PI3K/AKT/NF-κB pathway. The levels of PI3K p85, phosphorylated AKT-ser308, and NF-κB p65 were found to be positively correlated with TNC levels and were co-localized in prostate cancer tissues (Qian et al., 2022).

#### 3.3.3 TNC splicing regulation and pH balance in TME

Research has uncovered a significant link between the pH balance in TME and TNC splicing regulation. In normal human lung and skin fibroblasts, short isoforms lacking variable-splicing FNIII domains are predominantly expressed at a physiological pH of approximately 7.0. However, when the pH increases to 7.30–7.50 (as observed in fetal tissues and malignant tumors), TNC expression shifts towards long isoforms containing one or more variable-spliced FNIII domains (Giblin and Midwood, 2015). Notably, malignantly transformed fibroblasts (CAFs) mainly express long TNC isoforms, regardless of the extracellular pH. This is because these cells maintain an alkaline intracellular pH under various external conditions (Giblin and Midwood, 2015). This phenomenon may explain how CAFs, the primary source of TNC in TME, continue to produce long TNC isoforms that are more closely associated with the malignant environment, even in an acidic TME characterized by general hypoxia and lactate accumulation (Jin and Jin, 2020).

Despite the growing body of research linking TNC to tumor hypoxia, glycolytic reprogramming, and TME pH regulation, several key limitations persist. First, the majority of studies have primarily focused on the relationship between TNC and the HIF pathway, with insufficient attention given to its role in regulating other metabolic processes, such as lipid and amino acid metabolism. Second, current studies often provide only preliminary insights into the molecular mechanisms governing TNC’s interactions within the metabolic microenvironment. To address these gaps, future research should adopt a broader approach to more comprehensively elucidate the regulatory mechanisms by which TNC influences the tumor metabolic microenvironment.

### 3.4 TNC and RME

Our research team introduced the concept of RME as a novel aspect of TME. This concept seeks to explain how radiation therapy (RT), the primary treatment for many solid tumors, changes TME, and offers insights into improving clinical outcomes. We divided RME into two components: the radioimmune microenvironment and the radiation-hypoxic microenvironment (Zhu et al., 2023). Previously, we explored the relationships between TNC and various TMEs, as well as the connections between RME and other aspects of TME, such as the mechanical, metabolic, and immune microenvironments.

A previous study demonstrated that reducing TNC overexpression in radiation-resistant nasopharyngeal carcinoma cells (CNE-2R) increases their radiosensitivity (Liu et al., 2021). Similarly, Hsieh et al. found that in esophageal squamous cell carcinoma (ESCC), reduced expression of plasma gelsolin (pGSN) impairs its ability to compete with oncogenic TNC for binding to integrin αvβ3. This, in turn, activates TNC and promotes the formation of cancer-associated fibroblasts (CAFs), contributing to the development of radiotherapy resistance in tumors (Hsieh et al., 2024). TNC, a key regulator in RME, affects the mechanical microenvironment by enhancing MMP secretion (Cai et al., 2017; Wu et al., 2023), activating transformation-related transcription factors (Wu et al., 2023), and suppressing CAF apoptosis (Ni et al., 2017). It also impacts the immune microenvironment through interactions with integrin receptors α5β1 and αvβ6 (Jachetti et al., 2015; Mirzaei et al., 2018), and by stimulating the release of pro-inflammatory factors, such as TNF-α (Ma et al., 2019). Additionally, TNC influences the metabolic microenvironment through its significant association with the HIF signaling pathway (Xing et al., 2015; Zhang et al., 2022). These TNC-related factors in TME collectively shape RME. RME was further developed using a TNC lens. It should be noted that research on the relationship between TNC and RME is still in its infancy, with limited studies available. The specific mechanisms remain unclear and require further elucidation. Research on TNC and RME remains a promising area with significant potential for future investigation.

## 4 Summary and outlook

**FIGURE 1:**
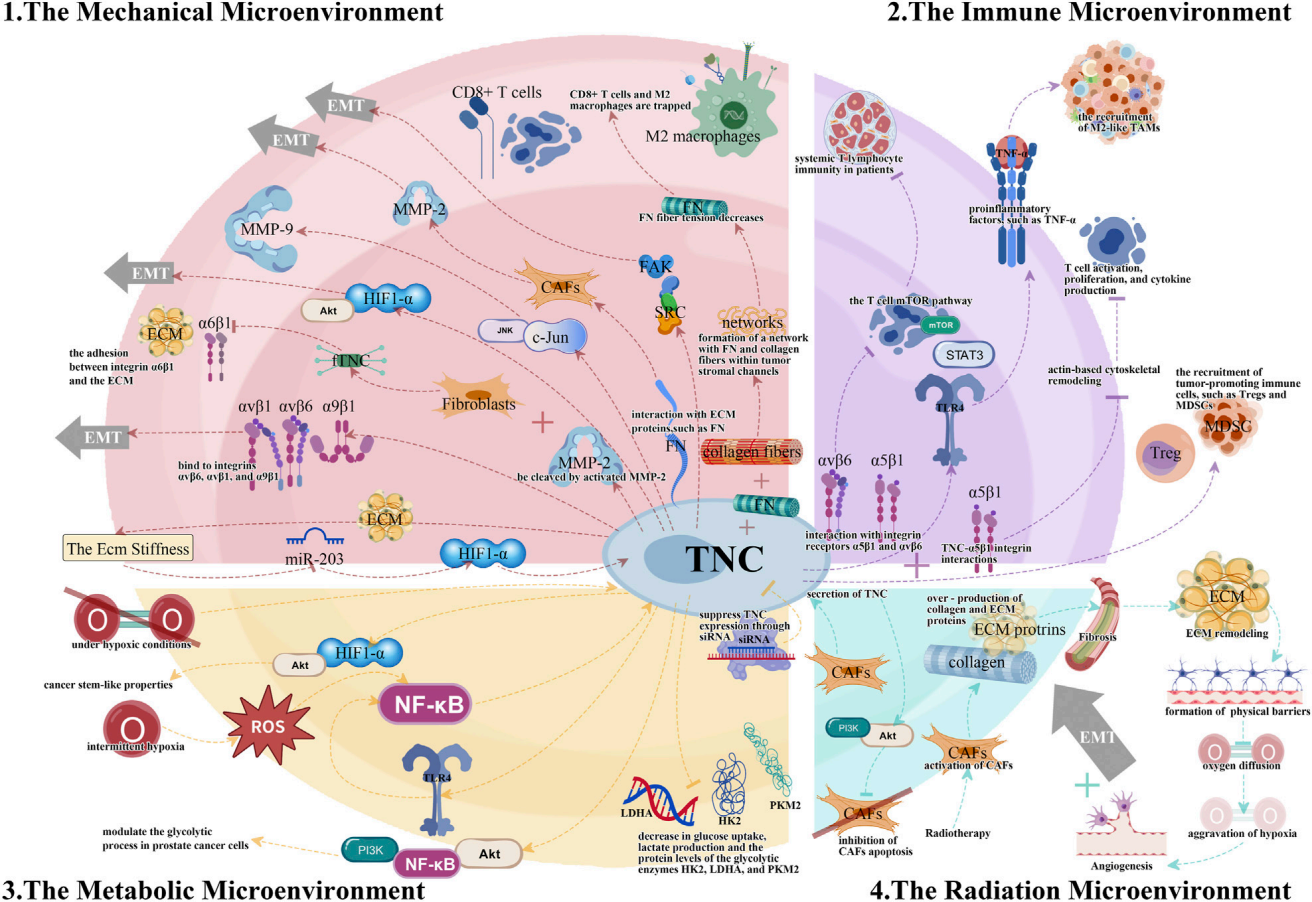
The different tumor microenvironments (TMEs) and the essential TNC signaling pathways.

The complexity and diversity of TME present major obstacles to cancer treatment. TNC, an important extracellular matrix component, regulates TME through various pathways and plays a vital role in tumor initiation and progression. This article provides a systematic review of research progress on TNC in mechanical, immune, and metabolic microenvironments, as well as RME, explaining the molecular mechanisms by which TNC promotes tumor progression through ECM remodeling, immune suppression, and metabolic reprogramming maintenance (Figure 1).

TNC is an important biomarker for predicting tumor infiltration and metastasis in various malignancies (Nong et al., 2015). In pancreatic cancer, the co-expression of the long isoform of TNC with membrane-associated annexin A2 (ANXA2) (Hagiwara et al., 2020) and the co-expression of TNC with MMP9 are significant indicators of poor patient prognosis (Cai et al., 2017). In LGGs, TNC can not only be used to assess the immunosuppressive microenvironment status and predict patient outcomes, but can also serve as a biomarker for immunotherapy efficacy prediction (Zhang et al., 2022). In prostate cancer, the TNC expression level can function as a potential biomarker for CAFs and is strongly associated with a poor patient prognosis (Ni et al., 2017). These findings lay the groundwork for the clinical application of TNC as a prognostic and therapeutic biomarker for tumors.

Research on TNC and tumors has primarily examined the connection between TNC and the mechanical microenvironment or the tumor ECM. Additional studies are needed to explore its relationship with other TMEs, particularly RME, which shows considerable promise for future research.

Emerging technologies, such as single-cell sequencing and spatial omics, are expected to shed light on the spatiotemporal dynamics of TNC regulation in TME. These methods allow the examination of TNC expression heterogeneity and its functional importance at the cellular level, mapping the spatial distribution of TNC in tumor tissues to comprehend its interactions with various microenvironmental elements, and monitoring dynamic changes in TNC expression levels to uncover its regulatory mechanisms during different phases of tumor progression. Furthermore, multi-omics integrative analysis can help decipher TNC-mediated signaling networks, laying the groundwork for innovative therapeutic approaches.

As a factor that promotes tumor growth, TNC is crucial for both fundamental and clinical research. In mechanistic research, focus should be placed on creating specific TNC inhibitors or blockers to offer new targeted therapy strategies; examining the distinct roles of various TNC isoforms in tumor progression, especially the unique functions of the long isoform of TNC in TME; investigating the interaction networks between TNC and other microenvironmental factors to reveal their collaborative mechanisms in tumor progression; and further clarifying the role of TNC in the radiotherapy microenvironment to provide new insights for enhancing radiotherapy effectiveness. In clinical research, clinical trials of TNC-targeted therapies should be conducted to assess their safety and efficacy; to explore the combined use of TNC with existing treatments, such as immune checkpoint inhibitors and radiotherapy; and to investigate the predictive value of TNC expression levels for treatment responses to inform personalized treatment plans.

In summary, a thorough understanding of how TNC functions in various TMEs will not only contribute to elucidating the molecular mechanisms of tumor progression, but will also offer novel approaches for personalized cancer therapy. Future studies should concentrate on exploring the synergistic interactions between TNC and other microenvironmental factors, developing more effective therapeutic strategies, and establishing more precise prognostic evaluation systems to improve cancer treatment outcomes.

## References

[B1] Brösicke N. Faissner A. (2015). Role of tenascins in the ECM of gliomas. Cell Adhesion and Migr. 9 (1-2), 131–140. 10.1080/19336918.2014.1000071 PMC4422794 25695402

[B2] Cai J. Du S. X. Wang H. Xin B. B. Wang J. Shen W. Y. (2017). Tenascin-C induces migration and invasion through JNK/c-Jun signalling in pancreatic cancer. Oncotarget 8 (43), 74406–74422. 10.18632/oncotarget.20160 29088796 PMC5650351

[B3] Cai X. J. Han M. Y. Lou F. Z. Sun Y. Yin Q. Q. Sun L. B. (2023). Tenascin C papillary fibroblasts facilitate neuro-immune interaction in a mouse model of psoriasis. Nat. Commun. 14 (1), 15. 10.1038/s41467-023-37798-x 37037861 PMC10086024

[B4] Chen J. Chen Z. Y. Chen M. Li D. J. Li Z. H. Xiong Y. (2009). Role of fibrillar Tenascin-C in metastatic pancreatic cancer. Int. J. Oncol. 34 (4), 1029–1036. 10.3892/ijo_00000228 19287959

[B5] Chen W. J. Wu Y. D. Wang J. Yu W. P. Shen X. Zhao K. (2024). Clinical advances in TNC delivery vectors and their conjugate agents. Pharmacol. and Ther. 253, 108577. 10.1016/j.pharmthera.2023.108577 38081519

[B6] Cheng X. Li F. Tao Z. Z. (2021). Tenascin-C promotes epithelial-to-mesenchymal transition and the mTOR signaling pathway in nasopharyngeal carcinoma. Oncol. Lett. 22 (1), 570. 10.3892/ol.2021.12831 34113398 PMC8185706

[B7] Dhaouadi S. Bouhaouala-Zahar B. Orend G. (2024). Tenascin-C targeting strategies in cancer. Matrix Biol. 130, 1–19. 10.1016/j.matbio.2024.04.002 38642843

[B8] Donovan C. Bai X. Chan Y. L. Feng M. Ho K. F. Guo H. (2023). Tenascin C in lung diseases. Biology-Basel 12 (2), 199. 10.3390/biology12020199 36829478 PMC9953172

[B9] Fonta C. M. Loustau T. Li C. B. Surendran S. P. Hansen U. Murdamoothoo D. (2023). Infiltrating CD8+T cells and M2 macrophages are retained in tumor matrix tracks enriched in low tension fibronectin fibers. Matrix Biol. 116, 1–27. 10.1016/j.matbio.2023.01.002 36669744

[B10] Fujita M. Sasada M. Eguchi M. Iyoda T. Okuyama S. Osawa T. (2021a). Induction of cellular senescence in fibroblasts through β1-integrin activation by tenascin-C-derived peptide and its protumor effect. Am. J. Cancer Res. 11 (9), 4364–4379. 34659892 PMC8493383

[B11] Fujita M. Suzuki H. Fukai F. (2021b). Involvement of integrin-activating peptides derived from tenascin-C in colon cancer progression. World J. Gastrointest. Oncol. 13 (9), 980–994. 10.4251/wjgo.v13.i9.980 34616507 PMC8465449

[B12] Furuhashi S. Morita Y. Matsumoto A. Ida S. Muraki R. Kitajima R. (2023). Tenascin C in pancreatic cancer-associated fibroblasts enhances epithelial mesenchymal transition and is associated with resistance to immune checkpoint inhibitor. Am. J. Cancer Res. 13 (11), 5641–5655. 38058842 PMC10695794

[B13] Gabrilovich D. I. Nagaraj S. (2009). Myeloid-derived suppressor cells as regulators of the immune system. Nat. Rev. Immunol. 9 (3), 162–174. 10.1038/nri2506 19197294 PMC2828349

[B14] Gao Z. H. Chen C. Gu P. Chen J. H. Liu X. D. Shen J. H. (2022). The tumor microenvironment and prognostic role of autophagy- and immune-related genes in bladder cancer. Cancer Biomarkers 35 (3), 293–303. 10.3233/cbm-220058 36245371 PMC12364238

[B15] Giblin S. P. Midwood K. S. (2015). Tenascin-C: form versus function. Cell Adhesion and Migr. 9 (1-2), 48–82. 10.4161/19336918.2014.987587 PMC4422809 25482829

[B16] Gutsche K. Randi E. B. Blank V. Fink D. Wenger R. H. Leo C. (2016). Intermittent hypoxia confers pro-metastatic gene expression selectively through NF-κB in inflammatory breast cancer cells. Free Radic. Biol. Med. 101, 129–142. 10.1016/j.freeradbiomed.2016.10.002 27717868

[B17] Guttery D. S. Hancox R. A. Mulligan K. T. Hughes S. Lambe S. M. Pringle J. H. (2010). Association of invasion-promoting tenascin-C additional domains with breast cancers in young women. Breast Cancer Res. 12 (4), R57. 10.1186/bcr2618 20678196 PMC2949648

[B18] Hagiwara K. Harimoto N. Yokobori T. Muranushi R. Hoshino K. Gantumur D. (2020). High Co-expression of large tenascin C splice variants in stromal tissue and annexin A2 in cancer cell membranes is associated with poor prognosis in pancreatic cancer. Ann. Surg. Oncol. 27 (3), 924–930. 10.1245/s10434-019-07708-x 31463696

[B19] Hancox R. A. Allen M. D. Holliday D. L. Edwards D. R. Pennington C. J. Guttery D. S. (2009). Tumour-associated tenascin-C isoforms promote breast cancer cell invasion and growth by matrix metalloproteinase-dependent and independent mechanisms. Breast Cancer Res. 11 (2), R24. 10.1186/bcr2251 19405959 PMC2688953

[B20] Hsieh C. H. Ho P. S. Wang W. L. Shih F. H. Hong C. T. Wang P. W. (2024). Decreased plasma gelsolin fosters a fibrotic tumor microenvironment and promotes chemoradiotherapy resistance in esophageal squamous cell carcinoma. J. Biomed. Sci. 31 (1), 90. 10.1186/s12929-024-01078-7 39261905 PMC11389350

[B21] Huang T. Lu C. Zhang Y. Lin B. Y. Zhang Z. J. Zhu D. (2023). Effect of activating cancer-associated fibroblasts biomarker TNC on immune cell infiltration and prognosis in breast cancer. Ann. Med. 55 (2), 2250987. 10.1080/07853890.2023.2250987 38375814 PMC10629425

[B22] Izumi K. Miyazaki N. Okada H. Tsujimoto A. Matsumoto-Miyazaki J. Naito J. (2020). Tenascin-C expression in renal biopsies from patients with tubulointerstitial nephritis and its relation to disease activity and prognosis. Int. J. Clin. Exp. Pathology 13 (7), 1842–1852. PMC7414498 32782713

[B23] Jachetti E. Caputo S. Mazzoleni S. Brambillasca C. S. Parigi S. M. Grioni M. (2015). Tenascin-C protects cancer stem-like cells from immune surveillance by arresting T-cell activation. Cancer Res. 75 (10), 2095–2108. 10.1158/0008-5472.Can-14-2346 25808872

[B24] Jarosz-Biej M. Smolarczyk R. Cichon T. Kulach N. (2019). Tumor microenvironment as A “game changer” in cancer radiotherapy. Int. J. Mol. Sci. 20 (13), 3212. 10.3390/ijms20133212 31261963 PMC6650939

[B25] Jin M. Z. Jin W. L. (2020). The updated landscape of tumor microenvironment and drug repurposing. Signal Transduct. Target. Ther. 5 (1), 166. 10.1038/s41392-020-00280-x 32843638 PMC7447642

[B26] Kang X. Xu E. Wang X. Z. Qian L. L. Yang Z. Yu H. (2021). Tenascin-c knockdown suppresses vasculogenic mimicry of gastric cancer by inhibiting ERK- triggered EMT. Cell Death and Dis. 12 (10), 890. 10.1038/s41419-021-04153-1 PMC8481562 34588421

[B27] Katoh D. Nagaharu K. Shimojo N. Hanamura N. Yamashita M. Kozuka Y. (2013). Binding of αvβ1 and αvβ6 integrins to tenascin-C induces epithelial-mesenchymal transition-like change of breast cancer cells. Oncogenesis 2, e65. 10.1038/oncsis.2013.27 23958855 PMC3759126

[B28] Labedz N. Anisiewicz A. Stachowicz-Suhs M. Banach J. Klopotowska D. Maciejczyk A. (2024). Dual effect of vitamin D on breast cancer-associated fibroblasts. Bmc Cancer 24 (1), 25. 10.1186/s12885-024-11961-z 38360633 PMC10868064

[B29] Lau K. M. Cheng S. H. Lo K. W. Lee S. Woo J. K. S. van Hasselt C. A. (2007). Increase in circulating Foxp3+CD4+CD25 high regulatory T cells in nasopharyngeal carcinoma patients. Br. J. Cancer 96 (4), 617–622. 10.1038/sj.bjc.6603580 17262084 PMC2360054

[B30] Lei X. Lei Y. Li J. K. Du W. X. Li R. G. Yang J. (2020). Immune cells within the tumor microenvironment: biological functions and roles in cancer immunotherapy. Cancer Lett. 470, 126–133. 10.1016/j.canlet.2019.11.009 31730903

[B31] Liu S. Y. Wang Z. Y. Zhu D. Q. Yang J. B. Lou D. D. Gao R. J. (2021). Effect of Shengmai Yin on the DNA methylation status of nasopharyngeal carcinoma cell and its radioresistant strains. J. Pharm. Analysis 11 (6), 783–790. 10.1016/j.jpha.2020.11.010 PMC8740367 35028184

[B32] Lowy C. M. Oskarsson T. (2015). Tenascin C in metastasis: a view from the invasive front. Cell Adhesion and Migr. 9 (1-2), 112–124. 10.1080/19336918.2015.1008331 PMC4422797 25738825

[B33] Ma D. Liu S. Q. Lal B. Wei S. Wang S. Y. Zhan D. Q. (2019). Extracellular matrix protein tenascin C increases phagocytosis mediated by CD47 loss of function in glioblastoma. Cancer Res. 79 (10), 2697–2708. 10.1158/0008-5472.Can-18-3125 30898840 PMC8218246

[B34] Miroshnikova Y. A. Mouw J. K. Barnes J. M. Pickup M. W. Lakins J. N. Kim Y. (2016). Tissue mechanics promote IDH1-dependent HIF1α-tenascin C feedback to regulate glioblastoma aggression. Nat. Cell Biol. 18(12)**,** 1336–1345. 10.1038/ncb3429 27820599 PMC5361403

[B35] Mirzaei R. Sarkar S. Dzikowski L. Rawji K. S. Khan L. Faissner A. (2018). Brain tumor-initiating cells export tenascin-C associated with exosomes to suppress T cell activity. Oncoimmunology 7 (10), e1478647. 10.1080/2162402x.2018.1478647 30288344 PMC6169571

[B36] Mitamura Y. Reiger M. Kim J. Xiao Y. Zhakparov D. Tan G. (2023). Spatial transcriptomics combined with single-cell RNA-sequencing unravels the complex inflammatory cell network in atopic dermatitis. Allergy 78 (8), 2215–2231. 10.1111/all.15781 37312623

[B37] Nagaharu K. Zhang X. H. Yoshida T. Katoh D. Hanamura N. Kozuka Y. (2011). Tenascin C induces epithelial-mesenchymal transition-like change accompanied by SRC activation and focal adhesion kinase phosphorylation in human breast cancer cells. Am. J. Pathology 178 (2), 754–763. 10.1016/j.ajpath.2010.10.015 PMC3069868 21281808

[B38] Ni W. D. Yang Z. T. Cui C. A. Cui Y. Fang L. Y. Xuan Y. H. (2017). Tenascin-C is a potential cancer-associated fibroblasts marker and predicts poor prognosis in prostate cancer. Biochem. Biophysical Res. Commun. 486 (3), 607–612. 10.1016/j.bbrc.2017.03.021 28341124

[B39] Ning L. G. Li S. Gao J. G. Ding L. Wang C. H. Chen W. G. (2019). Tenascin-C is increased in inflammatory bowel disease and is associated with response to infliximab therapy. Biomed Res. Int. 9, 1475705. 10.1155/2019/1475705 PMC6893280 31886172

[B40] Nong Y. H. Wu D. B. Lin Y. Zhang Y. Q. Bai L. Tang H. (2015). Tenascin-C expression is associated with poor prognosis in hepatocellular carcinoma (HCC) patients and the inflammatory cytokine TNF-α-induced TNC expression promotes migration in HCC cells. Am. J. Cancer Res. 5 (2), 782–791. 25973315 PMC4396033

[B41] Persa E. Balogh A. Sáfrány G. Lumniczky K. (2015). The effect of ionizing radiation on regulatory T cells in health and disease. Cancer Lett. 368 (2), 252–261. 10.1016/j.canlet.2015.03.003 25754816

[B42] Qian Y. R. Liu X. Z. Feng Y. Li X. G. Xuan Y. H. (2022). Tenascin C regulates cancer cell glycolysis and tumor progression in prostate cancer. Int. J. Urology 29 (6), 578–585. 10.1111/iju.14830 35218089

[B43] Shevtsov M. Sato H. Multhoff G. Shibata A. (2019). Novel approaches to improve the efficacy of immuno-radiotherapy. Front. Oncol. 9, 156. 10.3389/fonc.2019.00156 30941308 PMC6433964

[B44] Spenlé C. Loustau T. Murdamoothoo D. Erne W. Divonne S. B. D. Veber R. (2020). Tenascin-C orchestrates an immune-suppressive tumor microenvironment in oral squamous cell carcinoma. Cancer Immunol. Res. 8 (9), 1122–1138. 10.1158/2326-6066.Cir-20-0074 32665262

[B45] Steitz A. M. Steffes A. Finkernagel F. Unger A. Sommerfeld L. Jansen J. M. (2020). Tumor-associated macrophages promote ovarian cancer cell migration by secreting transforming growth factor beta induced (TGFBI) and tenascin C. Cell Death and Dis. 11 (4), 249. 10.1038/s41419-020-2438-8 PMC7171168 32312959

[B46] Telarovic I. Wenger R. H. Pruschy M. (2021). Interfering with tumor hypoxia for radiotherapy optimization. J. Exp. and Clin. Cancer Res. 40 (1), 197. 10.1186/s13046-021-02000-x 34154610 PMC8215813

[B47] Wang Y. Li Y. Q. Zhong J. P. Li M. Zhou Y. J. Lin Q. (2023). Tumor-derived Cav-1 promotes pre-metastatic niche formation and lung metastasis in breast cancer. Theranostics 13 (5), 1684–1697. 10.7150/thno.79250 37056561 PMC10086203

[B48] Wang Z. H. Tang Y. Tan Y. N. Wei Q. C. Yu W. (2019). Cancer-associated fibroblasts in radiotherapy: challenges and new opportunities. Cell Commun. Signal. 17, 47. 10.1186/s12964-019-0362-2 31101063 PMC6525365

[B49] Wickman E. Lange S. Wagner J. Ibanez J. Tian L. Q. Lu M. F. (2024). IL-18R supported CAR T cells targeting oncofetal tenascin C for the immunotherapy of pediatric sarcoma and brain tumors. J. Immunother. Cancer 12 (11), e009743. 10.1136/jitc-2024-009743 39572158 PMC11580246

[B50] Wu Y. Y. Hsu Y. L. Huang Y. C. Su Y. C. Wu K. L. Chang C. Y. (2023). Characterization of the pleural microenvironment niche and cancer transition using single-cell RNA sequencing in EGFR-mutated lung cancer. Theranostics 13 (13), 4412–4429. 10.7150/thno.85084 37649596 PMC10465223

[B51] Xing S. Q. Zhang C. G. Yuan J. F. Yang H. M. Zhao S. D. Zhang H. (2015). Adiponectin induces apoptosis in hepatocellular carcinoma through differential modulation of thioredoxin proteins. Biochem. Pharmacol. 93 (2), 221–231. 10.1016/j.bcp.2014.12.001 25514170

[B52] Yang Z. T. Zhang C. Y. Feng Y. Qi W. B. Cui Y. Xuan Y. H. (2019). Tenascin-C is involved in promotion of cancer sternness via the Akt/HIF1α axis in esophageal squamous cell carcinoma. Exp. Mol. Pathology 109, 61–68. 10.1016/j.yexmp.2019.03.007 30904401

[B53] Yilmaz A. Loustau T. Salomé N. Surendran S. P. Li C. B. Tucker R. P. (2022). Advances on the roles of tenascin-C in cancer. J. Cell Sci. 135 (18), jcs260244. 10.1242/jcs.260244 36102918 PMC9584351

[B54] Yoshida T. Akatsuka T. Imanaka-Yoshida K. (2015). Tenascin-C and integrins in cancer. Cell Adhesion and Migr. 9 (1-2), 96–104. 10.1080/19336918.2015.1008332 PMC4422796 25793576

[B55] Zhang P. Liu G. H. Hu J. Y. Chen S. Wang B. F. Peng P. (2022). Tenascin-C can serve as an indicator for the immunosuppressive microenvironment of diffuse low-grade gliomas. Front. Immunol. 13, 824586. 10.3389/fimmu.2022.824586 35371015 PMC8966496

[B56] Zhou S. A. Zhang W. Cai G. H. Ding Y. Z. Wei C. X. Li S. (2020). Myofiber necroptosis promotes muscle stem cell proliferation via releasing Tenascin-C during regeneration. Cell Res. 30 (12), 1063–1077. 10.1038/s41422-020-00393-6 32839552 PMC7784988

[B57] Zhu D. Q. Su C. Li J. J. Li A. W. Luv Y. Fan Q. (2023). Update on radiotherapy changes of nasopharyngeal carcinoma tumor microenvironment. World J. Oncol. 14 (5), 350–357. 10.14740/wjon1645 37869238 PMC10588496

